# *Parvimonas micra* and central nervous system infections: mechanisms of transformation from oral commensal to intracranial pathogen and clinical evidence

**DOI:** 10.3389/fcimb.2026.1971569

**Published:** 2026-10-05

**Authors:** Cancan Gong, Xubo Jing, Shanwei Jiang, Chaoxiao Yu

**Affiliations:** Department of Infectious Diseases, Yantaishan Hospital, Yantai, Shandong, China

**Keywords:** anaerobe, brain abscess, metagenomic next-generation sequencing, odontogenic infection, *Parvimonas micra*, pathobiont

## Abstract

*Parvimonas micra* (*P. micra*), an anaerobic oral coccus long dismissed as a culture contaminant, is increasingly recognized as a causative agent of pyogenic central nervous system (CNS) infections, yet its neuropathogenesis remains poorly defined. This narrative review synthesizes 41 peer-reviewed studies identified via a structured comprehensive literature search of PubMed, Embase and Web of Science through June 2026. Epidemiologically, pooling available case reports and small series (n=64 confirmed CNS cases), *P. micra* is among the most frequently isolated Gram-positive anaerobic cocci in reported brain abscess series, with proportions ranging from approximately 4% to over 10% in individual cohorts; species-level data are inconsistently reported and a precise pooled estimate cannot be derived. A male predominance (approximately 2.7:1) is observed among reported cases, though this should be interpreted cautiously given the small sample size and potential reporting bias. Approximately two-thirds of patients have no documented immunosuppressive condition. Brain abscess represents ~80% of CNS manifestations, with 60–70% presenting as polymicrobial infection. We propose a five-step pathogenic cascade—periodontal dysbiosis, epithelial barrier breach, intravascular survival, blood–brain barrier (BBB) translocation, and intracranial suppuration—as a conceptual framework. Virulence factors including the TmpC–AppA axis and extracellular vesicle DnaK have been characterized in non-CNS models (oral carcinoma, periodontitis, oral lichen planus); their relevance to CNS infection is inferred, and BBB translocation mechanisms remain entirely hypothetical. Metagenomic next-generation sequencing (mNGS) outperforms conventional culture in selected brain abscess cohorts (~85% vs ~50% in pus; ~84% vs ~8% in CSF across CNS infections), though performance varies by specimen type, prior antibiotic exposure, and sequencing platform. All-cause mortality is 5–10% and 20–25% of survivors sustain neurological sequelae, though these estimates may overestimate true population rates due to publication bias. *P. micra* shows consistently low *in vitro* minimum inhibitory concentrations to metronidazole and β-lactams. Early empirical anaerobic coverage and consideration of mNGS as a complementary diagnostic tool are recommended for suspected odontogenic brain abscess.

## 1 Introduction

Bacterial brain abscess remains a life-threatening infectious complication, with mortality persisting at 10–30% despite modern antimicrobial therapy and image-guided neurosurgery (Sonneville et al., 2017; Bodilsen et al., 2024). The traditional pathogenic paradigm—contiguous spread from sinonasal or otic foci, or haematogenous seeding from distant sites—has been substantially revised over the past decade. The oral microbiota is now recognized as an underrecognized reservoir of intracranial pathogens. Among neuroinvasive oral bacteria, *Parvimonas micra* has emerged as a clinically significant organism: long dismissed as a harmless commensal or incidental contaminant, it is now increasingly identified as a cause of brain abscess, meningitis, and intracranial vascular infections (Prieto et al., 2020; Takahashi et al., 2020; Shimizu et al., 2022; Chen et al., 2023).

Three parallel advances motivate this focused synthesis. First, clinical adoption of metagenomic next-generation sequencing (mNGS) has substantially improved detection of fastidious anaerobes, revealing that culture-based surveillance underestimated *P. micra* prevalence in CNS specimens (Qu et al., 2022; Wang et al., 2024). Second, periodontal biology studies have characterized the organism’s molecular virulence arsenal—including adhesins, invasins, and secreted effectors—that enable systemic dissemination from gingival niches (Ji et al., 2025; Qi et al., 2025; Li et al., 2026). Third, accumulating clinical data show *P. micra* CNS infections frequently occur in immunocompetent hosts without overt dental risk factors, challenging prevailing assumptions about opportunistic anaerobic pathogenesis. Although prior reviews have addressed *P. micra* bacteremia (Shimizu et al., 2022) and individual brain abscess case series (Chen et al., 2023), no CNS-focused review has systematically integrated recent molecular virulence discoveries with clinical diagnostic and therapeutic evidence, leaving clinicians without a unified framework.

*P. micra* (formerly *Peptostreptococcus micros*) is a Gram-positive, non-spore-forming anaerobic coccus colonizing gingival crevices, dental plaque, and tonsillar crypts in healthy adults (Higashi et al., 2023). Its extraoral clinical spectrum includes bacteremia, vertebral osteomyelitis, infective endocarditis, hepatic abscess, and intracranial suppuration (Gomez et al., 2015; Kim et al., 2019; García-Hita et al., 2020; Watanabe et al., 2020; Cesta et al., 2021; Suzuki et al., 2021; Aziz et al., 2024; Fu et al., 2024); fatal sepsis has also been reported, confirming it is not uniformly indolent (Jiang et al., 2022). This review consolidates current evidence on *P. micra* CNS infections, proposes a structured pathogenic cascade from oral commensal to intracranial pathogen as a conceptual framework, and summarizes current diagnostic and therapeutic strategies.

## 2 Search methodology and evidence quality

This work is a narrative review underpinned by a comprehensive structured literature search strategy; it was not registered on PROSPERO and does not include quantitative meta-analysis. The structured search was employed to maximize comprehensiveness and reproducibility, while the narrative synthesis format was selected given the heterogeneity of available evidence (predominantly case reports and small series with no comparator groups).

We conducted a comprehensive structured search of PubMed, Embase, and Web of Science from database inception through 15 June 2026. The PubMed search string was: (“*Parvimonas micra*”[Title/Abstract] OR “*Peptostreptococcus micros*”[Title/Abstract]) AND (“brain abscess”[MeSH Terms] OR “brain abscess”[Title/Abstract] OR “meningitis”[MeSH Terms] OR “meningitis”[Title/Abstract] OR “ventriculitis”[Title/Abstract] OR “intracranial suppuration”[Title/Abstract] OR “central nervous system infection”[Title/Abstract] OR “cerebrospinal fluid infection”[Title/Abstract]). Of note, “*Parvimonas micra*” is not indexed as a MeSH term in PubMed; all species-specific records were therefore identified via Title/Abstract searching. Embase and Web of Science searches used equivalent EMTREE and topic terms, respectively.

Eligible sources were peer-reviewed English full-text articles reporting clinically or microbiologically confirmed *P. micra* CNS infection in humans, or investigating *P. micra* virulence mechanisms relevant to invasive disease. Conference abstracts, non-original review articles, and studies failing to achieve species-level identification were excluded. Two reviewers (CG, XJ) independently screened titles and abstracts; discrepancies were resolved by consensus with a third reviewer (CY). The initial database search yielded approximately 80 records across the three databases; after deduplication and title/abstract screening, 26 records underwent full-text review, of which 17 primary clinical studies met inclusion criteria. Because *P. micra* virulence mechanisms have been characterized exclusively in non-CNS models (oral carcinoma, periodontitis, oral lichen planus), these mechanistic studies were not captured by the CNS-focused search string; accordingly, 7 mechanistic studies and 8 additional clinical studies were identified via citation tracking of included articles and relevant reviews. Together with 9 background references identified through the same process, this yielded 41 total sources for qualitative synthesis (25 clinical, 7 mechanistic, 9 background). A simplified PRISMA-style flow diagram summarizing the search and selection process is provided in **Supplementary Figure S1**.

Evidence quality was appraised using the Grading of Recommendations Assessment, Development and Evaluation (GRADE) framework across six core domains. Full evidence profiles, including risk of bias and indirectness ratings, are summarized in **Table 1**. Overall, epidemiological and therapeutic evidence was rated low to very low, limited by observational study design and publication bias; diagnostic evidence comparing mNGS to conventional culture was rated moderate; mechanistic evidence ranged from low (validated invasive pathways in non-CNS models) to very low (hypothesized BBB translocation mechanisms).

**Table 1:** GRADE evidence profile for key questions addressed in the review.

Key question	Study design	Risk of bias	Inconsistency	Indirectness	Imprecision	Publication bias	Overall quality
Epidemiology and clinical presentation of *P. micra* CNS infection	Case reports/series	Serious	Serious	Not serious	Serious	Likely	⊕⊝⊝⊝ Low
mNGS vs culture for diagnosis	Retrospective cohorts	Not serious	Not serious	Serious	Not serious	Undetected	⊕⊕⊕⊝ Moderate
Antimicrobial susceptibility	*In vitro* surveillance	Not serious	Not serious	Serious	Not serious	Undetected	⊕⊕⊝⊝ Low–moderate
TmpC–AppA invasive module	Experimental (oral carcinoma/periodontitis models)	Not serious	Not serious	Very serious	Serious	Undetected	⊕⊕⊝⊝ Low
BBB translocation mechanisms	Mechanistic (non-CNS models)	Serious	Serious	Very serious	Serious	Undetected	⊕⊝⊝⊝ Very low
Treatment regimens and outcomes	Case series/extrapolation	Serious	Serious	Not serious	Serious	Likely	⊕⊝⊝⊝ Very low

BBB, blood–brain barrier; GRADE, Grading of Recommendations Assessment, Development and Evaluation; mNGS, metagenomic next-generation sequencing; MIC, minimum inhibitory concentration.

## 3 The oral niche: from commensal carriage to invasive pathobiont

[Evidence: Established in oral models] In oral homeostasis, *P. micra* resides in subgingival biofilms, participating in syntrophic metabolic networks with *Fusobacterium nucleatum*, a bridging organism that mediates coaggregation and substantially increases *P. micra* biofilm biomass *in vitro* (Lamont and Hajishengallis, 2015; Horiuchi et al., 2020). The transition to pathogenic carriage begins with periodontal dysbiosis: periodontitis deepens gingival pockets, lowers local oxidation-reduction potential, and enriches proteolytic anaerobes, with *P. micra* loads significantly higher at diseased sites (Higashi et al., 2023). As a pathobiont, its pathogenic potential is unmasked by local ecological disruption rather than acquisition of exogenous virulent clones (Lamont and Hajishengallis, 2015; Higashi et al., 2023). The organism actively drives tissue destruction via strain-variable collagenase, elastase, and hemolytic activity (Ota-Tsuzuki and Alves Mayer, 2010).

[Evidence: Demonstrated in non-CNS models; relevance to CNS inferred] Three interconnected virulence modules support invasive capacity. First, the TmpC–AppA axis mediates host cell invasion and intracellular survival: the surface lipoprotein TmpC binds cytoskeleton-associated protein 4 (CKAP4) on epithelial and endothelial cells, triggering clathrin-dependent endocytosis (Qi et al., 2025). The secreted effector AppA enables resistance to lysosomal acidification and degradation, establishing an intracellular replicative niche (Li et al., 2026). These mechanisms have been characterized in oral squamous cell carcinoma and periodontitis models; their relevance to CNS infection is inferred from the expression of CKAP4 and integrin αvβ3 on cerebral endothelial cells, but they have not been demonstrated in CNS endothelial cells or *in vivo* CNS infection models. Second, extracellular vesicle (EV)-delivered DnaK engages the host BAG3–IKK-γ–NF-κB axis in oral fibroblasts, driving TNF-α secretion and contact-independent inflammation (Ji et al., 2025). DnaK should be regarded as a potential inflammatory mediator rather than an established virulence factor for CNS disease: direct evidence that *P. micra* EV-DnaK contributes to CNS invasion, immune evasion, or intracranial tissue damage is lacking, and the study was conducted in oral lichen planus fibroblasts, not CNS cells. Third, cell wall lipoteichoic acid activates TLR2 on myeloid cells, eliciting proinflammatory cytokine release, while secreted proteases degrade extracellular matrix to facilitate tissue spread (Higashi et al., 2023; Ji et al., 2025). Additional pathways characterized in cancer models represent plausible but unproven immune evasion mechanisms unvalidated in invasive infection (Bergsten et al., 2023; Chang et al., 2023).

[Evidence: Established clinically] For systemic invasion, *P. micra* must first breach the oral epithelial barrier. Dental procedures (extraction, scaling, root canal therapy) are well-documented precipitants of bacteremia, but even routine toothbrushing can induce low-level bacteremia in patients with gingival inflammation (Watanabe et al., 2020; Shimizu et al., 2022). In severe periodontitis, ulcerated pocket epithelium may reach 10–20 cm², providing a substantial portal of entry even without recent dental intervention (Higashi et al., 2023), explaining why many CNS cases lack identifiable dental sources (Shimizu et al., 2022; Chen et al., 2023).

## 4 Haematogenous dissemination and blood–brain barrier translocation

[Evidence: Inferred from non-CNS models and clinical association] Bacteremia is a necessary but insufficient prerequisite for brain abscess formation. As an obligate anaerobe, *P. micra* relies on three complementary adaptations to survive systemic circulation: upregulation of oxidative stress responses, microcolony formation with hypoxic inner cores, and platelet-associated transit (Sonneville et al., 2017; Higashi et al., 2023). Microcolonies in plasma not only generate a self-sustaining anaerobic microenvironment, but also evade neutrophil engulfment; association with fibrin and platelets further provides immunological camouflage and contributes to thromboembolic complications (Horiuchi et al., 2020; Higashi et al., 2023; Aziz et al., 2024; Fu et al., 2024; Sak et al., 2024). The organism also evades innate immunity via peptidoglycan modifications, recruitment of host complement regulators, and proteolytic cleavage of complement components and immunoglobulins (Higashi et al., 2023). These adaptations are supported by *in vitro* data and documented *P. micra* bacteremia cases, but intravascular survival specifically in the context of CNS dissemination has not been directly demonstrated.

[Evidence: Hypothetical] The blood–brain barrier (BBB) represents the principal anatomical barrier to haematogenous CNS infection. No study has directly examined *P. micra* BBB traversal, and all proposed routes are hypothesized by analogy with better-characterized pathogens. Three non-mutually exclusive potential pathways are proposed: (1) transcellular invasion via the TmpC–CKAP4 axis, biologically plausible given CKAP4 expression on cerebral endothelium and TmpC’s established role in epithelial internalization (Qi et al., 2025; Li et al., 2026); (2) paracellular penetration via bacterial proteases or host-derived matrix metalloproteinases disrupting tight junctions (Ota-Tsuzuki and Alves Mayer, 2010); and (3) Trojan-horse transit via infected phagocytes, which remains unvalidated as *P. micra* myeloid intracellular survival has not been demonstrated. Indirect evidence supporting the hypothesis of BBB translocation includes: (a) direct isolation of *P. micra* from brain abscess pus and CSF, demonstrating that the organism reaches the CNS compartment; (b) temporal association between documented *P. micra* bacteremia and subsequent CNS infection in several case reports (Watanabe et al., 2020; Aziz et al., 2024; Fu et al., 2024); and (c) demonstrated BBB translocation by the closely related oral anaerobe *Fusobacterium nucleatum* via the FadA–VE-cadherin pathway (Fardini et al., 2011), providing a mechanistic precedent. Specific experiments required to establish CNS invasion include: *in vitro* BBB models using human brain microvascular endothelial cells (hBMECs) in transwell systems; confocal microscopy to distinguish transcellular from paracellular transit; murine bacteremia models with quantitative CNS tissue culture and histopathology; and transcriptomic analysis of *P. micra* recovered from brain tissue versus oral isolates to identify CNS-adaptive gene expression.

[Evidence: Established in periodontal disease; inferred in CNS infection] A fourth complementary mechanism involves polymicrobial interactions. In periodontal disease, *Fusobacterium nucleatum* has been demonstrated to form synergistic biofilms with *P. micra in vitro*, with increased biomass and enhanced antimicrobial resistance (Horiuchi et al., 2020). In CNS infection, *F. nucleatum* is frequently co-isolated with *P. micra* in brain abscesses (Takahashi et al., 2020; Wang et al., 2024), and expresses FadA adhesin that binds VE-cadherin and disrupts endothelial junctional integrity (Fardini et al., 2011). It is hypothesized that *F. nucleatum* may act as a pioneer pathogen, opening paracellular routes for subsequent *P. micra* penetration (Lamont and Hajishengallis, 2015; Takahashi et al., 2020; Wang et al., 2024). However, it is important to note that no study has demonstrated synergistic virulence between *P. micra* and *F. nucleatum* in a CNS infection model. The high proportion of polymicrobial brain abscesses may alternatively reflect the polymicrobial nature of the oral inoculum and shared oral source, rather than true cooperative pathogenicity within the CNS. The clinical significance of co-isolation remains uncertain. Strategies to disrupt polymicrobial oral biofilms, including cyanobacterial fatty acid esters with demonstrated activity against oral biofilm consortia, represent an active area of investigation (Bharathidasan et al., 2026).

[Evidence: Established clinically] Once within brain parenchyma, *P. micra* pathogen-associated molecular patterns engage TLR2 on microglia and myeloid cells, activating NF-κB signaling and driving the classic four-stage brain abscess progression (early cerebritis, late cerebritis, capsule formation, mature abscess) (Sonneville et al., 2017; Bodilsen et al., 2024). The hypoxic, acidic abscess core is highly permissive for *P. micra* growth but blunts neutrophil oxidative killing; the fibrous capsule also creates a pharmacological sanctuary, necessitating prolonged antibiotic therapy and surgical drainage for sizable lesions (Sonneville et al., 2017; Bodilsen et al., 2024).

The proposed five-step pathogenic cascade of *Parvimonas micra* from oral commensal to intracranial pathogen is summarized in **Figure 1** as a conceptual framework: (1) periodontal dysbiosis with synergistic biofilm formation with *F. nucleatum* [Established in oral models]; (2) epithelial barrier breach via the TmpC–AppA invasive axis [Demonstrated in non-CNS models; relevance to CNS inferred]; (3) intravascular survival through microcolony formation, complement evasion, and platelet association [Inferred from non-CNS models and clinical association]; (4) BBB translocation via transcellular, paracellular, or Trojan-horse routes [Hypothetical]; (5) intracranial suppuration with abscess capsule formation [Established clinically]. Steps with direct experimental or clinical evidence are indicated by solid arrows; inferred or hypothetical steps are shown with dashed arrows.

**Figure 1:**
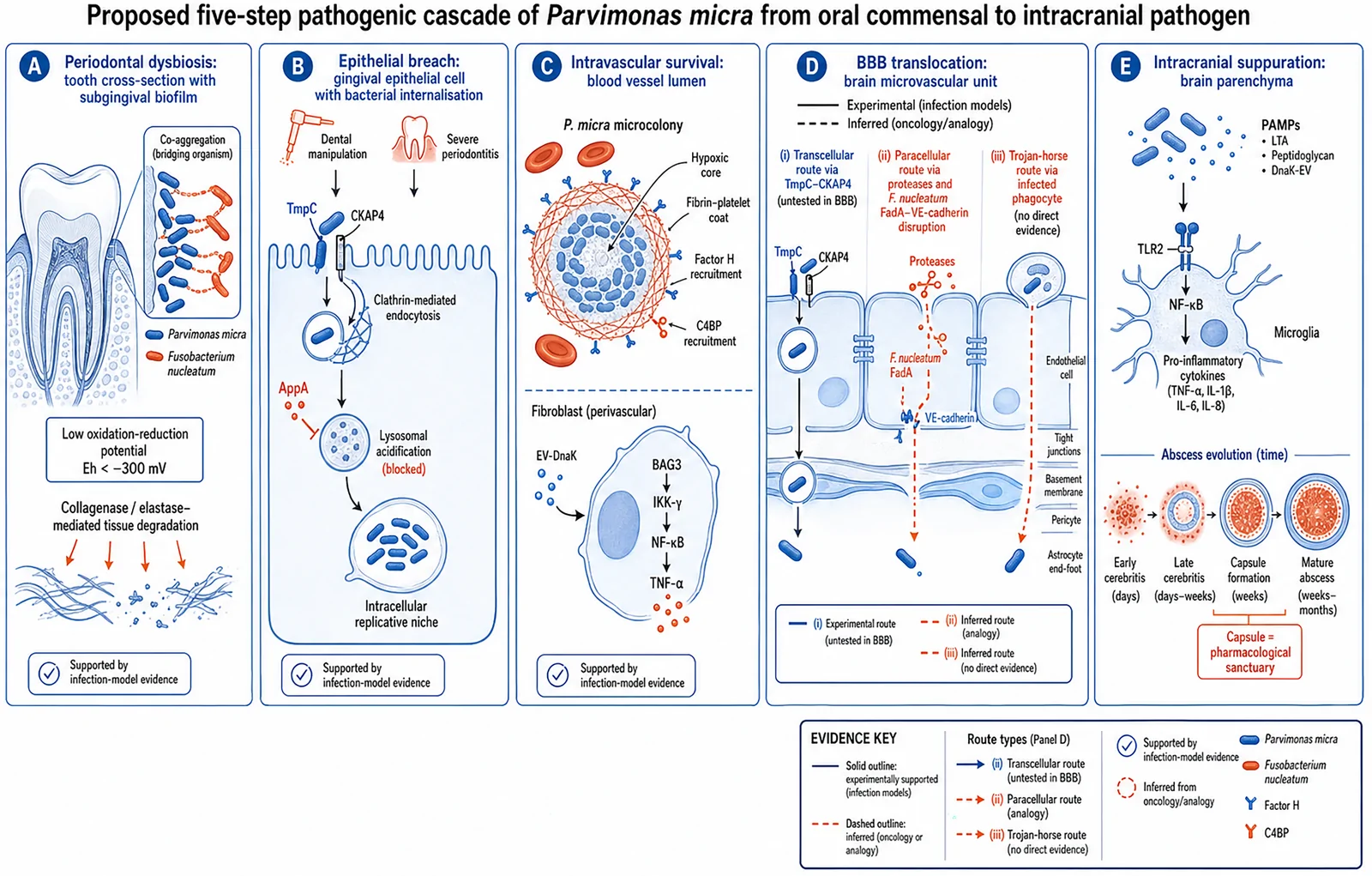
Five-step pathogenic cascade of *Parvimonas micra*. Proposed five-step pathogenic cascade of *Parvimonas micra* from oral commensal to intracranial pathogen. **(A)** Periodontal dysbiosis promotes anaerobic, proteolytic biofilm enrichment and co-aggregation with *Fusobacterium nucleatum*. **(B)** Epithelial breach is illustrated by TmpC-associated internalization, AppA-linked lysosomal evasion, and bacteremia triggered by dental manipulation or severe periodontitis. **(C)** Intravascular persistence is represented by microcolony formation, fibrin–platelet shielding, complement regulator recruitment, and EV-DnaK signaling to fibroblasts through BAG3–IKK-γ–NF-κB with TNF-α output. **(D)** Candidate BBB routes include transcellular entry, paracellular passage, and Trojan-horse dissemination; inferred routes are visually separated from experimentally supported elements. **(E)** Intracranial inflammation culminates in staged abscess evolution and capsule formation, creating a pharmacological sanctuary. AppA, acid phosphatase A (lysosomal evasion effector); BAG3, Bcl-2-associated athanogene 3; BBB, blood–brain barrier; CKAP4, cytoskeleton-associated protein 4; Eh, oxidation–reduction potential; EV, extracellular vesicle; IKK-γ, inhibitor of nuclear factor κ-B kinase subunit gamma; LTA, lipoteichoic acid; NF-κB, nuclear factor-κB; PAMP, pathogen-associated molecular pattern; TLR2, Toll-like receptor 2; TmpC, trigger of myeloid cells (surface lipoprotein); TNF, tumor necrosis factor; VE-cadherin, vascular endothelial cadherin.

## 5 Clinical epidemiology and disease spectrum

All epidemiological estimates are derived predominantly from published case reports and small retrospective case series, subject to significant publication bias favoring severe presentations; true population-level incidence of *P. micra* CNS infection remains undefined. To permit transparent appraisal, we pooled individual patient-level data from the 12 studies reporting discrete CNS cases (refs (Ko et al., 2015; Shtaya et al., 2017; Kim et al., 2019; Ochi et al., 2020; Prieto et al., 2020; Takahashi et al., 2020; Cesta et al., 2021; Jiang et al., 2022; Shimizu et al., 2022; Chen et al., 2023; Sak et al., 2024; Andrés-Eslava et al., 2026)), yielding 64 patients with confirmed *P. micra* CNS infection for descriptive analysis. Of these, 52 had partial or complete individual-level data available; the remaining 12 were derived from aggregate-only series and are noted as such. Where individual-level data were unavailable, estimates are taken directly from the source publication. Key clinical studies spanning the spectrum of disease presentations are summarized in **Table 2**.

**Table 2:** Key clinical studies of *Parvimonas micra* central nervous system infection.

Study	Year	n (CNS)	CNS syndrome(s)	Micro. diagnosis	Mono/Poly	Presumed source	Therapy & outcome
Shimizu et al. (2022)	2022	26 (bacteremia; CNS subset)	Brain abscess, meningitis	Culture + 16S PCR	Mixed (60% poly)	Dental (periodontitis)	Metronidazole + ceftriaxone; favorable
Chen et al. (2023)	2023	15 (pooled)	Brain abscess	Culture + mNGS	Poly (73%)	Odontogenic (60%)	Drainage + IV antibiotics; mortality 6.7%
Wang et al. (2024)	2024	12 *P. micra*/85 brain abscess	Brain abscess (poly)	mNGS (85% vs 50% culture)	Poly	Odontogenic	mNGS-guided therapy; improved detection
Cesta et al. (2021)	2021	1	Liver + brain abscess	Culture + MALDI-TOF	Mono	Haematogenous (GI)	Metronidazole + ceftriaxone; recovery
Prieto et al. (2020)	2020	3	Odontogenic brain abscess	Culture + 16S rRNA	Poly (2/3)	Dental (all)	Drainage + antibiotics; full recovery
Takahashi et al. (2020)	2020	1	Brain abscess	Culture + 16S PCR	Poly (A. cardiffensis)	Odontogenic	Drainage + meropenem; recovery
Andrés-Eslava et al. (2026)	2026	1	Brain abscess + meningitis	mNGS + culture	Mono	Idiopathic (immunocompetent)	Ceftriaxone + metronidazole; recovery
Ko et al. (2015)	2015	1	Bacteraemic meningitis	Blood culture (CSF neg)	Mono	Odontogenic	Penicillin G; full recovery
Sak et al. (2024)	2024	1	Orbital cellulitis + CVT	Culture + mNGS	Poly	Dental	Anticoagulation + antibiotics; neuro deficit
Shtaya et al. (2017)	2017	1	Cervical + brain abscess	Culture + 16S	Mono	Esophageal pleural fistula	Drainage + antibiotics; recovery
Jiang et al. (2022)	2022	1	Fatal sepsis + CNS invasion	Blood culture + mNGS	Mono	Periodontal	Multidrug regimen; fatal (fulminant)
Ochi et al. (2020)	2020	1 (pediatric)	Brain abscess	Culture + 16S PCR	Poly	Intraoral trauma	Drainage + antibiotics; recovery
Pooled (this review)	2026	64 (confirmed)	Brain abscess 80%, meningitis 12%	Culture 50–70%, mNGS 85%	Poly 60–70%	Dental 60%, idiopathic 30%	Mortality 5–10%; sequelae 20–25%

Studies were selected to represent the range of clinical syndromes, diagnostic modalities and outcomes. mNGS-based studies are over-represented in recent years, reflecting the diagnostic shift described in the text. The final row presents pooled individual patient-level data from 12 studies reporting discrete CNS cases (n=64); percentages are descriptive and subject to publication bias. n, number; mNGS, metagenomic next-generation sequencing; CVT, cerebral venous thrombosis; CSF, cerebrospinal fluid.

The apparent increase in diagnosed *P. micra* CNS infections over the past decade largely reflects diagnostic advances, as mNGS has increased anaerobic bacterial detection in brain abscess cohorts by 3–5-fold compared with culture (Qu et al., 2022; Wang et al., 2024). Anaerobes account for ~10% of all brain abscess cases. Regarding the proportion attributable specifically to *P. micra*: no published systematic review reports a precise species-level percentage. Available data indicate that *P. micra* is among the most frequently isolated Gram-positive anaerobic cocci in reported brain abscess series, with proportions ranging from approximately 4% in an Indian polymicrobial series (Chowdhury et al., as cited in (Urbán and Gajdács, 2021)) to over 10% in individual cohorts. In a Hungarian retrospective cohort of 64 brain abscesses, *P. micra* was among the most commonly isolated Gram-positive anaerobic cocci, alongside Finegoldia magna and Peptostreptococcus anaerobius, though a specific percentage was not reported (Urbán and Gajdács, 2021). In a systematic review of 28 studies encompassing 715 anaerobic brain abscess cases, Gram-positive anaerobic cocci collectively accounted for 35.1% of isolates, but species-level data for *P. micra* were not separately reported (Cobo, 2023). Species-level data are inconsistently reported across studies, and a precise pooled estimate cannot be derived from the available literature.

Clinically, a male predominance (approximately 2.7:1 male-to-female ratio) is observed among reported cases, potentially partially explained by higher severe periodontitis prevalence in males (Watanabe et al., 2020; Shimizu et al., 2022; Chen et al., 2023). However, this ratio should be interpreted cautiously given the small pooled sample (n=64), the absence of population-level denominator data, and potential reporting bias; whether this reflects true biological susceptibility, differences in oral health-seeking behavior, or ascertainment bias remains unknown. Approximately two-thirds of patients (42 of 64, where data were available) have no documented immunosuppressive condition, defined here as the absence of HIV infection, active malignancy, solid organ or haematopoietic stem cell transplant, long-term corticosteroid or immunosuppressant therapy, primary immunodeficiency, or uncontrolled diabetes mellitus. We note that this classification is based on information reported in the primary publications, not on formal immunological testing; subclinical immune deficits may therefore be underrecognized. Identifiable risk factors in the remaining third include diabetes mellitus, active malignancy, TNF-α inhibitor therapy, cyanotic congenital heart disease, and prosthetic heart valves (García-Hita et al., 2020; Lo et al., 2020; Shimizu et al., 2022; Chen et al., 2023; Fu et al., 2024; Andrés-Eslava et al., 2026). A substantial minority of cases lack an identifiable dental source, with rare non-odontogenic portals such as esophageal pleural fistula documented (Shtaya et al., 2017; Shimizu et al., 2022; Chen et al., 2023).

Brain abscess accounts for ~80% of *P. micra* CNS disease, typically presenting subacutely with headache (70–80%), focal neurological deficits (50–60%), and fever (40–50%) (Prieto et al., 2020; Takahashi et al., 2020; Shimizu et al., 2022; Chen et al., 2023). Lesions predominate in the frontal and parietal lobes, with classic ring enhancement on contrast MRI and restricted diffusion on DWI (Prieto et al., 2020; Chen et al., 2023). Polymicrobial infection occurs in 60–70% of cases, most commonly with *F. nucleatum* and *Streptococcus anginosus* group species, with direct implications for antimicrobial regimen design (Takahashi et al., 2020; Al Saleemi et al., 2024; Eichorn et al., 2024; Wang et al., 2024). Pediatric cases have also been reported, almost exclusively in the setting of polymicrobial infection following intraoral trauma (Ochi et al., 2020). Less common manifestations include meningitis, cerebral venous thrombosis, infectious intracranial aneurysms, and intraventricular rupture with ventriculitis, all associated with higher morbidity (Ko et al., 2015; Sak et al., 2024).

### 5.1 Clinical suspicion in the absence of dental risk factors

Given that approximately one-third of reported *P. micra* CNS cases lack an identifiable dental source, clinicians should maintain a low threshold for considering anaerobic infection even without overt odontogenic risk factors. Several scenarios warrant particular vigilance. First, occult periodontal disease may not be apparent on routine examination, and comprehensive dental assessment including panoramic radiography or dental CT should be considered in culture-negative brain abscess of unclear source. Second, gastrointestinal sources have been documented, particularly in patients with recent abdominal surgery, inflammatory bowel disease, or esophageal pathology (Shtaya et al., 2017). Third, iatrogenic sources include recent dental procedures, endoscopy, and immunosuppressive therapy including TNF-α inhibitors (Lo et al., 2020). Fourth, dental implant surfaces and prosthetic materials may serve as niduses for anaerobic biofilm formation, and novel coating strategies are under investigation to reduce microbial colonization (Ganapathy et al., 2026). Fifth, cryptogenic cases in which no source is identified constitute a meaningful minority. In culture-negative brain abscess or meningitis, particularly when imaging suggests an odontogenic pattern (frontal or temporal lobe location, sinus involvement), clinicians should send specimens for anaerobic culture and mNGS even in the absence of a clear dental history.

## 6 Diagnosis

Diagnosis of *P. micra* CNS infection is constrained by the organism’s fastidious growth requirements. Strict anaerobic culture requires prereduced transport media and rapid inoculation, yet sensitivity for anaerobic brain abscess remains only 50–70%, with *P. micra* particularly prone to overgrowth by faster-proliferating taxa (Urbán and Gajdács, 2021; Cobo, 2023). Prior antimicrobial exposure further reduces yield, and historical phenotypic identification was frequently inaccurate, misclassifying *P. micra* as other anaerobic cocci (Qu et al., 2022; Wang et al., 2024).

mNGS has substantially altered diagnostic practice by detecting fastidious and antibiotic-suppressed organisms without culture reliance. In a retrospective cohort of 127 brain abscess specimens, Wang et al. (2024) reported an mNGS detection rate of approximately 85% in pus specimens versus 50% for conventional culture, with anaerobes detected 3–5-fold more frequently by mNGS. For cerebrospinal fluid, a systematic review and meta-analysis of CNS infections (encompassing viral, bacterial, and fungal etiologies) by Qu et al. (2022) found mNGS positivity of approximately 84% versus only approximately 8% for conventional culture. These performance estimates should be interpreted as reported values in selected cohorts rather than generalizable diagnostic accuracy metrics: diagnostic performance depends strongly on specimen type (pus is far more sensitive than CSF), prior antibiotic exposure (which suppresses culture but not mNGS), sequencing platform and bioinformatics pipeline, the choice of diagnostic reference standard (culture versus composite clinical diagnosis), and study population (tertiary referral centers may overrepresent complex cases). The CSF estimate in particular includes non-bacterial CNS infections and may overstate the culture deficit specific to anaerobic brain abscess. Additional advantages of mNGS include full polymicrobial community characterization and preserved performance despite prior antibiotic use; in multiple reports, mNGS was the sole modality identifying *P. micra* as the causative organism (Chen et al., 2022; Ma et al., 2024; Wang et al., 2024). Targeted next-generation sequencing panels offer lower cost and faster turnaround than untargeted mNGS, but cannot detect rare or unexpected pathogens, and their CNS performance requires further validation (Zhang et al., 2024).

mNGS should be considered as a complementary diagnostic tool, particularly in culture-negative cases, when conventional workup fails to identify a pathogen, or when anaerobic infection is suspected but culture is negative. Key practical limitations of mNGS include: (1) cost—currently $200–500 per test in most settings, not universally reimbursed; (2) turnaround time—24–72 hours, which may delay targeted therapy; (3) contamination—reagent and environmental contaminants are a particular problem for low-biomass CSF specimens; (4) low-abundance interpretation—distinguishing true pathogens from colonizers or contaminants requires clinical correlation and established read-count thresholds; (5) availability—largely restricted to tertiary centers and commercial laboratories; and (6) the need for clinical correlation—mNGS results must be interpreted alongside imaging, CSF profile, and epidemiological context. mNGS does not replace conventional culture and susceptibility testing. Results must be interpreted alongside clinical, radiological, and laboratory findings, including sequencing read depth and presence of corroborating oral taxa (Chen et al., 2022). 16S rRNA PCR and MALDI-TOF MS remain valuable confirmatory tools (Qu et al., 2022; Lars et al., 2023; Wang et al., 2024). For suspected anaerobic brain abscess, recommended workup includes contrast brain MRI with DWI, paired aerobic/anaerobic blood cultures, dental assessment, and stereotactic aspiration for lesions >2.5 cm, with pus submitted for simultaneous anaerobic culture, mNGS, and 16S rRNA PCR. A practical diagnostic and management algorithm for suspected odontogenic anaerobic brain abscess, incorporating mNGS testing and empirical antimicrobial recommendations, is provided in **Figure 2**.

**Figure 2:**
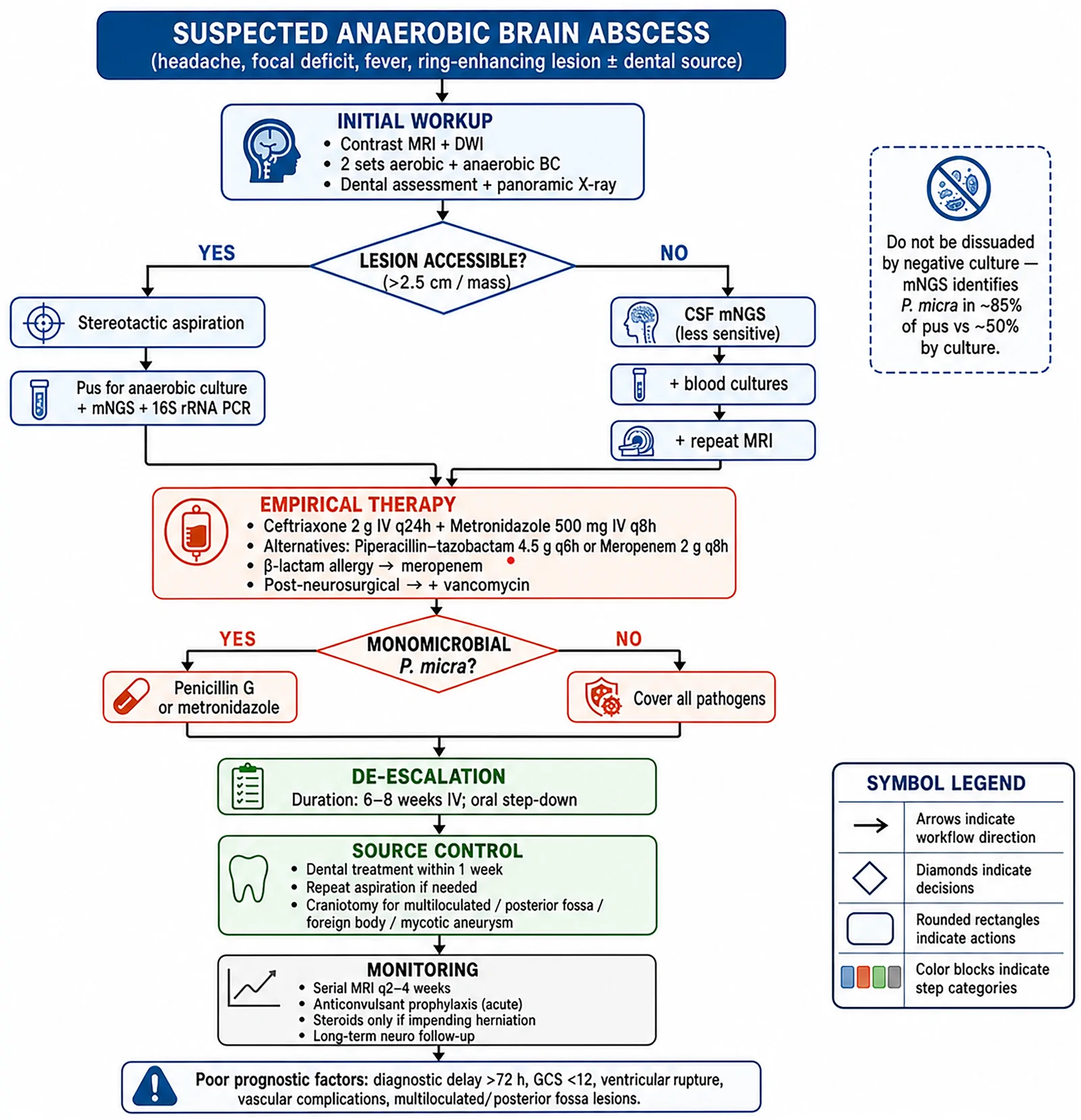
Diagnostic algorithm for odontogenic brain abscess. Practical diagnostic and management algorithm for suspected odontogenic anaerobic brain abscess, incorporating the role of mNGS and the typical antimicrobial susceptibility profile of *P. micra*. Empirical regimens are extrapolated from ESCMID brain abscess guidance and *in vitro* susceptibility data. Steroids are reserved for mass effect with impending herniation. De-escalation to monotherapy is appropriate only when *P. micra* is isolated in pure culture; polymicrobial infection requires coverage of all co-pathogens. BC, blood cultures; CNS, central nervous system; CSF, cerebrospinal fluid; DWI, diffusion-weighted imaging; GCS, Glasgow Coma Scale; IV, intravenous; mNGS, metagenomic next-generation sequencing; MRI, magnetic resonance imaging; PCR, polymerase chain reaction; q, every (quaque).

## 7 Treatment and outcomes

Management of *P. micra* CNS infection follows core principles for anaerobic brain abscess, with two key emphases: early dental evaluation and source control to prevent recurrence, and cautious de-escalation in polymicrobial cases, which must await full identification and susceptibility testing of all co-pathogens (Sonneville et al., 2017; Bodilsen et al., 2024). Treatment recommendations vary by infection type, as detailed below. All recommendations are extrapolated from general anaerobic CNS infection guidelines, as *P. micra*-specific therapeutic trials do not exist.

*P. micra* demonstrates consistently high *in vitro* susceptibility to first-line anti-anaerobic agents. *In vitro* susceptibility profiles, CNS pharmacokinetic properties, and recommended adult doses for principal agents are summarized in **Table 3**. The most robust susceptibility data come from Watanabe et al. (2020), who reported susceptibility rates among 25 *P. micra* bloodstream isolates: penicillin 100% (22/22), ampicillin–sulbactam 100% (12/12), piperacillin–tazobactam 100% (12/12), meropenem 100% (19/19), and clindamycin 86.7% (13/15); literature review cases showed similarly high susceptibility rates. Of note, metronidazole was not tested in that series. For CNS isolates specifically, Chen et al. (2023) reported an antibiogram from a confirmed *P. micra* brain abscess isolate demonstrating susceptibility to penicillin, piperacillin–tazobactam, cefmetazole, flomoxef, clindamycin, metronidazole, and chloramphenicol; their literature review of 15 *P. micra* brain abscess cases found metronidazole-based regimens in the majority of treated cases, with 90% of cases showing neurological improvement. Individual case reports have documented low minimum inhibitory concentrations (MICs) for clinical CNS isolates, but no large systematic surveillance study of *P. micra* antimicrobial susceptibility exists, and reported MIC values should be interpreted as preliminary estimates from small series. It is important to distinguish *in vitro* susceptibility from clinical efficacy: low MICs do not guarantee therapeutic success because CNS penetration, protein binding, abscess-pH effects, and biofilm growth can all reduce *in vivo* activity. Susceptibility data are limited to small series, no large surveillance study exists, and geographic and temporal variation may occur. EUCAST has not established *P. micra*-specific breakpoints, so interpretive criteria are extrapolated from anaerobic cocci group breakpoints. *P. micra* is not known to produce β-lactamase (Cobo, 2023; Bodilsen et al., 2024); however, because 60–70% of CNS cases are polymicrobial and may include β-lactamase-producing co-pathogens (e.g., Prevotella, *Fusobacterium* spp.), empirical monotherapy with narrow-spectrum penicillin is inappropriate until full susceptibility results are available. Metronidazole remains the cornerstone of anti-anaerobic therapy due to excellent CSF and abscess pus penetration, with >99% of *P. micra* isolates susceptible *in vitro* based on limited published data (Cobo, 2023; Bodilsen et al., 2024). Although a 3.2% rate of metronidazole treatment failure has been reported in a *P. micra* bacteremia series (Shimizu et al., 2022), no confirmed metronidazole-resistant *P. micra* isolates have been published to our knowledge; treatment failure may reflect inadequate CNS penetration, biofilm growth, or insufficient source control rather than microbiological resistance. Anaerobic susceptibility surveillance remains limited and emerging resistance cannot be excluded. Recommended empirical regimens include ceftriaxone plus metronidazole, piperacillin–tazobactam monotherapy, or meropenem for severe β-lactam allergy (Bodilsen et al., 2024). For confirmed monomicrobial *P. micra* infection with documented susceptibility, de-escalation to penicillin G or metronidazole monotherapy is reasonable. Clindamycin resistance affects 10–15% of contemporary isolates based on available series (Watanabe et al., 2020; Cobo, 2023), precluding its use as sole empirical therapy. Susceptibility testing is recommended for patients with prior prolonged anti-anaerobic exposure, persistent bacteremia, or treatment failure, consistent with ESCMID and IDSA guidance on anaerobic infections (Sonneville et al., 2017; Bodilsen et al., 2024); where direct *P. micra*-specific guidance is absent, this recommendation is extrapolated from general anaerobic infection principles.

**Table 3:** *In vitro* susceptibility and central nervous system pharmacokinetic profile of principal antimicrobials active against *Parvimonas micra*.

Antimicrobial	Susceptibility rate	Susceptibility	CSF penetration	Recommended dose (IV)	Role in *P. micra* CNS infection
Penicillin G	100% S (Watanabe et al., 2020)	100% S	Moderate (inflamed meninges)	20–24 MU/day divided q4–6h	Definitive monotherapy if susceptible; limited CNS penetration in non-inflamed meninges
Metronidazole	>99% S (limited data) (Cobo, 2023; Bodilsen et al., 2024)	>99% S	Excellent (several-fold above MIC)	500 mg q8h	Cornerstone; excellent abscess and CSF penetration; not tested in Watanabe series
Ceftriaxone	Good (streptococci; variable anaerobes)	Good (streptococci; variable anaerobes)	Good (inflamed meninges)	2 g q24h	Empirical partner with metronidazole for polymicrobial coverage
Piperacillin–tazobactam	100% S (Watanabe et al., 2020)	Susceptible	Good	4.5 g q6h	Empirical monotherapy alternative; covers β-lactamase producers
Meropenem	100% S (Watanabe et al., 2020)	Susceptible	Good	2 g q8h	β-lactam allergy or resistant co-pathogens
Clindamycin	86.7% S (13/15) (Watanabe et al., 2020); 10–15% resistant	85–90% S (10–15% resistant)	Good	600 mg q6h	Not for empirical monotherapy; use only if susceptibility confirmed
Vancomycin	Gram-positive cover	Gram-positive cover	Good (inflamed)	15 mg/kg q12h (trough 15–20)	Add if post-neurosurgical or staphylococcal concern

Susceptibility rates are compiled from Watanabe et al. (2020) (25 *P. micra* bloodstream isolates; penicillin, ampicillin–sulbactam, piperacillin–tazobactam, meropenem, clindamycin tested; metronidazole not included), Chen et al. (2023) (1 brain abscess isolate with full antibiogram: penicillin, piperacillin–tazobactam, cefmetazole, flomoxef, clindamycin, metronidazole, chloramphenicol all susceptible; 15-case literature review showing metronidazole use in majority of cases with 90% neurological improvement), Shimizu et al. (2022) (26 bacteremia cases; 3.2% metronidazole treatment failure reported without confirmed resistant isolates), the ESCMID brain abscess guideline (Bodilsen et al., 2024), and Cobo et al (Cobo, 2023). EUCAST has not established *P. micra*-specific breakpoints; interpretive criteria are extrapolated from anaerobic cocci group breakpoints. No large systematic surveillance study of *P. micra* antimicrobial susceptibility exists; individual MIC values reported in case series should be regarded as preliminary. *In vitro* susceptibility does not guarantee clinical efficacy due to variable CNS penetration, protein binding, and biofilm effects. No confirmed metronidazole-resistant *P. micra* isolates have been published; clindamycin resistance affects 10–15% of contemporary isolates. *P. micra* itself is generally beta-lactamase negative, but co-isolated species in polymicrobial infections may produce beta-lactamase. CNS, central nervous system; CSF, cerebrospinal fluid; MIC, minimum inhibitory concentration.

### 7.1 Infection-type-specific treatment recommendations

Brain abscess: 6–8 weeks of intravenous therapy (metronidazole plus third-generation cephalosporin), followed by 2–4 weeks of oral step-down if clinically improving; surgical drainage is standard for abscesses >2.5 cm. Meningitis: 4–6 weeks intravenous therapy, with emphasis on CSF-penetrating agents; metronidazole achieves adequate CSF levels in inflamed meninges. Subdural empyema: requires urgent surgical drainage plus 4–6 weeks intravenous antibiotics; medical management alone is inadequate. Epidural abscess: similar to subdural empyema, with surgical decompression and 4–6 weeks of therapy. Polymicrobial disease: regimen must cover all isolated organisms; broad empiric coverage (e.g., ceftriaxone plus metronidazole ± vancomycin) is recommended until culture and susceptibility results guide de-escalation.

### 7.2 Source control

Source control is as important as antimicrobial therapy in preventing treatment failure and relapse. Dental evaluation: all patients with suspected odontogenic CNS infection should receive comprehensive dental assessment, including panoramic radiography or dental CT, once acute infection is controlled. Periodontal treatment: definitive periodontal therapy (scaling, root planing, extraction of non-salvageable teeth) should be performed after stabilization, typically within 2–4 weeks of CNS infection diagnosis, to eliminate the presumed source and prevent recurrence. Dental implant surfaces and prosthetic materials may serve as niduses for anaerobic biofilm formation, and novel coating strategies are under investigation to reduce microbial colonization (Ganapathy et al., 2026). Brain abscess drainage: stereotactic needle aspiration is preferred for most abscesses >2.5 cm or with mass effect; open craniotomy is reserved for multiloculated or posterior fossa abscesses, and those associated with mycotic aneurysms (Sonneville et al., 2017; Bodilsen et al., 2024). Neurosurgical intervention timing: urgent drainage within 24 hours is indicated for abscesses with neurological deterioration, increased intracranial pressure, or intraventricular rupture. Adjunctive corticosteroids are reserved for impending herniation due to risks of impaired antimicrobial penetration and delayed capsule formation; anticonvulsant prophylaxis is reasonable for supratentorial abscesses during the acute phase (Sonneville et al., 2017; Bodilsen et al., 2024).

All-cause mortality for P. micra brain abscess is estimated at 5–10% based on pooled case data from the studies summarized in **Table 2** and systematic reviews of anaerobic brain abscess (Urbán and Gajdács, 2021; Chen et al., 2023; Cobo, 2023), lower than many other bacterial causes, consistent with its low intrinsic virulence and excellent antimicrobial susceptibility. Among the 15 P. micra brain abscess cases reviewed by Chen et al. (2023), 90% documented neurological improvement; in a separate bacteremia series by Shimizu et al. (2022), mortality was 3.7% (1/27), though this reflects bacteremia rather than CNS-specific outcomes. However, these estimates likely overestimate true population rates due to publication bias favoring severe cases, small sample sizes, and variable follow-up duration across reports. Approximately 20–25% of survivors sustain permanent neurological sequelae, defined here as persistent neurological deficit at last follow-up, including motor weakness, cognitive impairment, seizure disorder, visual disturbance, or aphasia (Urbán and Gajdács, 2021; Chen et al., 2023; Cobo, 2023). Follow-up duration ranges from 1 month to 5 years across reports, and some studies do not systematically report long-term outcomes. Poor prognostic factors include diagnostic delay >72 hours, Glasgow Coma Scale score <12 on admission, ventricular rupture, vascular complications, and multiloculated lesions. Omission of initial anaerobic coverage is also associated with worse outcomes. Clinicians should use these figures cautiously for individual prognosis, as mild or subclinical cases may be substantially underrepresented in the literature.

## 8 Limitations and future directions

This synthesis has several important limitations. First, study design: the clinical evidence base consists almost entirely of case reports and small retrospective series; no prospective cohorts, randomized trials, or controlled studies exist for *P. micra* CNS infection. Second, publication bias: severe and atypical cases are overrepresented in the literature, likely inflating estimated mortality and complication rates; the reported 5–10% mortality and 20–25% sequelae rates should therefore be regarded as upper-bound estimates. Third, mechanistic extrapolation: all virulence factor data derive from non-CNS models (oral carcinoma, periodontitis, oral lichen planus); no CNS-specific mechanistic studies exist, and BBB translocation mechanisms remain entirely hypothetical. Fourth, diagnostic heterogeneity: studies use varying culture methods, mNGS platforms, and diagnostic criteria, limiting cross-study comparison; the reported mNGS performance metrics reflect selected cohorts rather than generalizable accuracy. Fifth, treatment evidence: all treatment recommendations are extrapolated from general anaerobic CNS infection guidelines; no *P. micra*-specific therapeutic trials exist, and *in vitro* susceptibility does not guarantee clinical efficacy. Sixth, susceptibility testing: no standardized species-specific breakpoints exist (EUCAST has not established *P. micra* breakpoints), and surveillance data are limited to small series. Seventh, antimicrobial susceptibility data are limited: no large systematic surveillance study exists, most data derive from small bloodstream isolate series that do not test metronidazole, only one published CNS isolate has a documented full antibiogram (Chen et al., 2023), a 3.2% metronidazole treatment failure rate has been reported without confirmed microbiological resistance (Shimizu et al., 2022), and EUCAST has not established *P. micra*-specific breakpoints. Eighth, epidemiological imprecision: no precise pooled estimate of *P. micra*’s proportion among anaerobic brain abscesses can be derived, as species-level data are inconsistently reported across studies.

Three priority research gaps should be addressed: (1) functional validation of the TmpC–CKAP4 transcytosis hypothesis and polymicrobial BBB synergy in human cerebral endothelial cell transwell models and murine CNS infection models, including transcriptomic analysis of brain-derived versus oral *P. micra* isolates; (2) prospective multicenter studies to establish validated mNGS read-count thresholds for distinguishing invasive *P. micra* infection from contamination, and to define standardized diagnostic algorithms; (3) development of an international multicenter registry to enable robust epidemiological analysis, evaluation of standardized treatment regimens, and systematic surveillance of antimicrobial susceptibility patterns including emerging resistance.

## 9 Conclusions

*P. micra* has evolved from a dismissed culture contaminant to an increasingly recognized cause of CNS infection, particularly brain abscess. Its trajectory from oral commensal to intracranial pathogen can be conceptualized as a proposed five-step cascade, though BBB translocation mechanisms and neurotropism determinants remain largely hypothetical and require experimental validation. Recent molecular discoveries of the TmpC–AppA invasive axis and EV-DnaK inflammatory pathway—characterized in oral carcinoma, periodontitis, and oral lichen planus models—provide preliminary mechanistic hypotheses that require validation in CNS-relevant systems; these findings should not be interpreted as established CNS pathogenic mechanisms. mNGS has revealed the true disease burden while exposing the inadequacy of culture-based diagnostics, though performance varies by specimen type and clinical context, and mNGS should be regarded as a complementary rather than replacement tool. Outcomes are favorable with early anaerobic coverage, source control including dental evaluation and periodontal treatment, and timely surgical intervention. Targeted mechanistic research and multicenter clinical registration are needed to advance understanding of this underrecognized pathogen.
